# Assessing the Efficacy of ChatGPT Versus Human Researchers in Identifying Relevant Studies on mHealth Interventions for Improving Medication Adherence in Patients With Ischemic Stroke When Conducting Systematic Reviews: Comparative Analysis

**DOI:** 10.2196/51526

**Published:** 2024-05-06

**Authors:** Suebsarn Ruksakulpiwat, Lalipat Phianhasin, Chitchanok Benjasirisan, Kedong Ding, Anuoluwapo Ajibade, Ayanesh Kumar, Cassie Stewart

**Affiliations:** 1 Department of Medical Nursing, Faculty of Nursing, Mahidol University Bangkok Thailand; 2 Jack, Joseph and Morton Mandel School of Applied Social Sciences, Case Western Reserve University Cleveland, OH United States; 3 College of Art and Science, Department of Anthropology, Case Western Reserve University Cleveland, OH United States; 4 School of Medicine, Case Western Reserve University Cleveland, OH United States; 5 Frances Payne Bolton School of Nursing, Case Western Reserve University Cleveland, OH United States

**Keywords:** ChatGPT, systematic reviews, medication adherence, mobile health, mHealth, ischemic stroke, mobile phone

## Abstract

**Background:**

ChatGPT by OpenAI emerged as a potential tool for researchers, aiding in various aspects of research. One such application was the identification of relevant studies in systematic reviews. However, a comprehensive comparison of the efficacy of relevant study identification between human researchers and ChatGPT has not been conducted.

**Objective:**

This study aims to compare the efficacy of ChatGPT and human researchers in identifying relevant studies on medication adherence improvement using mobile health interventions in patients with ischemic stroke during systematic reviews.

**Methods:**

This study used the PRISMA (Preferred Reporting Items for Systematic Reviews and Meta-Analyses) guidelines. Four electronic databases, including CINAHL Plus with Full Text, Web of Science, PubMed, and MEDLINE, were searched to identify articles published from inception until 2023 using search terms based on MeSH (Medical Subject Headings) terms generated by human researchers versus ChatGPT. The authors independently screened the titles, abstracts, and full text of the studies identified through separate searches conducted by human researchers and ChatGPT. The comparison encompassed several aspects, including the ability to retrieve relevant studies, accuracy, efficiency, limitations, and challenges associated with each method.

**Results:**

A total of 6 articles identified through search terms generated by human researchers were included in the final analysis, of which 4 (67%) reported improvements in medication adherence after the intervention. However, 33% (2/6) of the included studies did not clearly state whether medication adherence improved after the intervention. A total of 10 studies were included based on search terms generated by ChatGPT, of which 6 (60%) overlapped with studies identified by human researchers. Regarding the impact of mobile health interventions on medication adherence, most included studies (8/10, 80%) based on search terms generated by ChatGPT reported improvements in medication adherence after the intervention. However, 20% (2/10) of the studies did not clearly state whether medication adherence improved after the intervention. The precision in accurately identifying relevant studies was higher in human researchers (0.86) than in ChatGPT (0.77). This is consistent with the percentage of relevance, where human researchers (9.8%) demonstrated a higher percentage of relevance than ChatGPT (3%). However, when considering the time required for both humans and ChatGPT to identify relevant studies, ChatGPT substantially outperformed human researchers as it took less time to identify relevant studies.

**Conclusions:**

Our comparative analysis highlighted the strengths and limitations of both approaches. Ultimately, the choice between human researchers and ChatGPT depends on the specific requirements and objectives of each review, but the collaborative synergy of both approaches holds the potential to advance evidence-based research and decision-making in the health care field.

## Introduction

### Background

Artificial intelligence (AI) is the field of computer science that studies and develops systems that can perform tasks, typically requiring human intelligence, such as reasoning, learning, decision-making, natural language processing (NLP), computer vision, and speech recognition [1]. AI is a rapidly evolving field with applications in various domains, for example, health care, education, business, and entertainment [2]. One of the subfields of AI is NLP, which deals with analyzing and generating natural language texts [3]. Chatbots, a type of NLP system, can interact with humans using natural language, either through text or speech. Chatbots can be used for various purposes, including customer service, entertainment, education, and information retrieval [3]. However, developing chatbots that can engage in natural and coherent conversations with humans is a challenging task that requires advanced NLP techniques and large-scale data.

One of the recent advances in NLP is the development of GPT models, which are neural network models that can generate natural language texts based on a given input or context [4]. GPT models are trained on large corpora of text from various sources, such as books, websites, news articles, and social media posts [4]. GPT models have been used to create chatbots that can generate realistic and diverse responses to human queries or messages [4]. Although GPT models have been developed by various research groups and companies (ie, OpenAI, Google, Facebook, and Microsoft), the first one was introduced by OpenAI in 2019 [5]. Since then, ChatGPT has been improved and refined by researchers and developers, who have applied it to various tasks and scenarios, such as customer service, education, entertainment, and social media [5]. ChatGPT models aim to provide engaging, informative, and coherent dialogues with users across different domains and tasks [4].

ChatGPT has been applied in the medical field in various ways. For instance, in medical practice, it has the ability to help streamline the clinical workflow, enhance diagnostics, and predict disease risk and outcome [6]. For medical education, ChatGPT can be useful in tailoring education and enabling powerful self-learning [6]. In terms of medical research, a previous study reported that ChatGPT has the potential to advance understanding, identify new research questions, and improve data analysis and interpretation [7]. In addition, ChatGPT extends to involve in writing articles through improvement in language and communication of result findings [6]. In particular, in the literature review process, which is time and effort consuming, ChatGPT has a promising advantage because of its potential ability to analyze large amounts of data, particularly in scientific articles [8]. Furthermore, ChatGPT was reported to have the potential to generate effective Boolean queries for systematic review literature searches [9].

Although ChatGPT has several advantages in medical research, it has limitations that could impact the quality of research, particularly in the literature review and search strategies processes. Citation inaccuracies, insufficient references, and references to nonexistent sources were reported as current problems [6]. Moreover, ChatGPT has a limited knowledge period based on the data sets used in ChatGPT training, which limits the reliability of the updated source of the literature review [6]. In a previous study, researchers were advised to consider the potential for incorrect MeSH (Medical Subject Headings) terms and the varying effectiveness of search queries with multiple requests when devising search strategies for a systematic review [9]. However, ChatGPT has a high potential to be used in medical research in the future. Therefore, it is imperative to explore and develop to improve and use it effectively.

Despite the significant benefits and limitations of using ChatGPT, the evaluation of the quality and performance of ChatGPT models in the review process remains unclear. Therefore, this study aims to compare the efficacy of ChatGPT and human researchers in identifying relevant health-related studies, such as research on medication adherence improvement using mobile health (mHealth) interventions in patients with ischemic stroke. The review will use systematic methods to search, select, appraise, and synthesize to address the following questions: (1) How does ChatGPT’s performance compare to that of human researchers in terms of accuracy in identifying relevant studies? (2) What challenges and limitations arise from using ChatGPT versus human researchers for identifying relevant studies in systematic reviews? (3) What are the implications of using ChatGPT to enhance the efficiency of systematic reviews? The results of this review will provide crucial insights into the potential of ChatGPT as an innovative tool for conducting systematic reviews.

### Objectives

This study aims to compare the efficacy of using ChatGPT and human researchers in identifying relevant studies on medication adherence improvement using mHealth interventions in patients with ischemic stroke during systematic reviews.

## Methods

### Identify Relevant Studies

In this study, we used the PRISMA (Preferred Reporting Items for Systematic Reviews and Meta-Analyses) [10] guidelines to identify the relevant studies. Overall, 4 electronic databases, including CINAHL Plus with Full Text, Web of Science, PubMed, and MEDLINE, were searched to identify articles published from inception until 2023 on using mHealth interventions for improving medication adherence in patients with ischemic stroke. We used search terms based on MeSH using Boolean phrases generated by human researchers and ChatGPT version 3.5 to identify relevant studies. The reference lists of the included studies, generated by human researchers and ChatGPT, were separately stored and screened in EndNote (EndNote X7 reference management software package). A PRISMA flow diagram was created to present the results of the search and screening process.

### Study Selection

The authors independently screened the titles and abstracts of the studies identified through separate searches conducted by human researchers and ChatGPT to determine their relevance. Subsequently, the full text of the selected articles was also assessed to ensure they met the predetermined inclusion criteria. A consistent set of inclusion criteria was applied to ensure that only studies relevant to the review’s objective were included. In contrast, the same exclusion criteria were used to eliminate literature unrelated to the review (Textbox 1).

**Study inclusion and exclusion criteria.**

**Inclusion criteria**

- Studies that aimed to use mobile health interventions for improving medication adherence
- Studies that primarily included adults with ischemic stroke or transient ischemic attack (TIA) aged ≥18 years (if the study included other stroke types, such as hemorrhagic stroke, it is acceptable, but the study population must include adults with ischemic stroke or TIA)
- Studies in English
- Studies that were published from inception until 2023

**Exclusion criteria**

- Studies that included children or adolescents aged <18 years
- Conference proceedings, abstracts, review articles, protocols, dissertations, letters to the editor, brief reports, or statement papers
- Studies that involved animal samples

### Data Extraction

A separate summary table for data extraction is presented in Multimedia Appendix 1 [11-20], consisting of the following data for each study: reference, year, country, study design, sample size, target population, intervention and objective, and main findings. This table will be used to compare the included studies obtained through the *Identify relevant studies* phase conducted by human researchers versus ChatGPT. The primary outcome of interest is medication adherence among patients with ischemic stroke. Medication adherence can be measured using various methods, such as drug level measurement, pill count, electronic databases, self-report questionnaires, and electronic monitoring systems [21]. The findings from studies that aimed to use mHealth interventions for improving medication adherence but did not measure medical adherence directly will be evaluated based on how they operationalized medication adherence according to their study design.

### Data Analysis

In this study, we will assess the accuracy of both human researchers and ChatGPT in identifying relevant studies from electronic databases by measuring precision. Precision is a performance metric that measures the accuracy of a model’s positive predictions. It focuses on the proportion of correctly identified positive instances (true positives) out of all the cases that the model predicted as positive (true positives+false positives) [22]. Precision is calculated using the following formula: precision=true positives/(true positives+false positives).

A high precision value close to 1 indicates that the model has a low rate of false positives. This means that when the model predicts an instance as positive, it will likely be correct. In contrast, a low precision value close to 0 indicates that the model has a high rate of false positives. This means that when the model predicts an instance as positive, it often needs to be corrected [22]. In the context of this study, precision will help evaluate the ability of both human researchers and ChatGPT to accurately identify relevant studies from electronic databases during the systematic review process. We will compare their precision scores to determine which approach yields a higher proportion of true positives and a lower rate of false positives.

In addition, as the human researcher will still need to conduct the screening, eligibility, and inclusion phases, we will also calculate the percentage of relevance using the formula ([true positives/total studies identified from the search]×100). This approach will be chosen to ensure a fair assessment, as relying solely on a formula based on true and false positives (precision) might only reflect human variability and accuracy during the screening, eligibility, and inclusion phases.

### Ethical Considerations

This study considers nonhuman research according to the “Self-Assessment form whether an activity is human subject research which requires ethical approval” recommended by Mahidol University Central Institutional Review Board. Therefore, ethics approval from the research ethics committee was not required.

## Results

### Search Term

#### Human Researcher

In the search phase, we used search terms based on MeSH using Boolean operators. The searched topic was related to using mHealth interventions for improving medication adherence in patients with ischemic stroke: (Ischemic Stroke* OR Cryptogenic Ischemic Stroke* OR Cryptogenic Stroke* OR Cryptogenic Embolism Stroke* OR Wake up Stroke* OR Acute Ischemic Stroke* OR Embolic Stroke* OR Cardioembolic Stroke* OR Cardioembolic Stroke* OR Thrombotic Stroke* OR Acute Thrombotic Stroke* OR Lacunar Stroke* OR Lacunar Syndrome* OR Lacunar Infarction* OR Lacunar Infarct*) AND (Medication Adherence OR Medication Nonadherence OR Medication Noncompliance OR Medication Persistence OR Medication Compliance OR Medication Non-Compliance) AND (Tele-Referral* OR Virtual Medicine OR Tele Intensive Care OR Tele ICU OR Mobile Health OR mHealth OR Telehealth OR eHealth OR Remote Consultation OR Teleconsultation* OR Telenursing OR Telepathology OR Teleradiology OR Telerehabilitation* OR Remote Rehabilitation* OR Virtual Rehabilitation*).

#### ChatGPT

To compare with the search by human researchers, we asked ChatGPT [23] on June 23, 2023, at 1:30 PM EST to provide a search term for conducting a systematic review of the same topic as follows: “Hello ChatGPT, we are researchers and currently conduct a systematic review titled: Using m-health interventions for improving medication adherence in ischemic stroke patients. Can you provide Medical Subject Headings (MeSH) search terms and combine them using Boolean operators for a search process?” The following search terms resulted from ChatGPT, which we used in the search phase and then compared the results with those from human researchers: (Mobile Applications OR Cell Phone OR Smartphone OR Telemedicine OR Text Messaging OR Internet) AND (Medication Adherence OR Patient Compliance OR Medication Systems, Intelligent) AND (Stroke OR Ischemic Attack, Transient OR Cerebrovascular Disorders). The search term (generated by human researchers and ChatGPT) was adjusted according to the database searching requirement before searching, but the original keyword was not changed.

### Search Results

**Figure 1:**
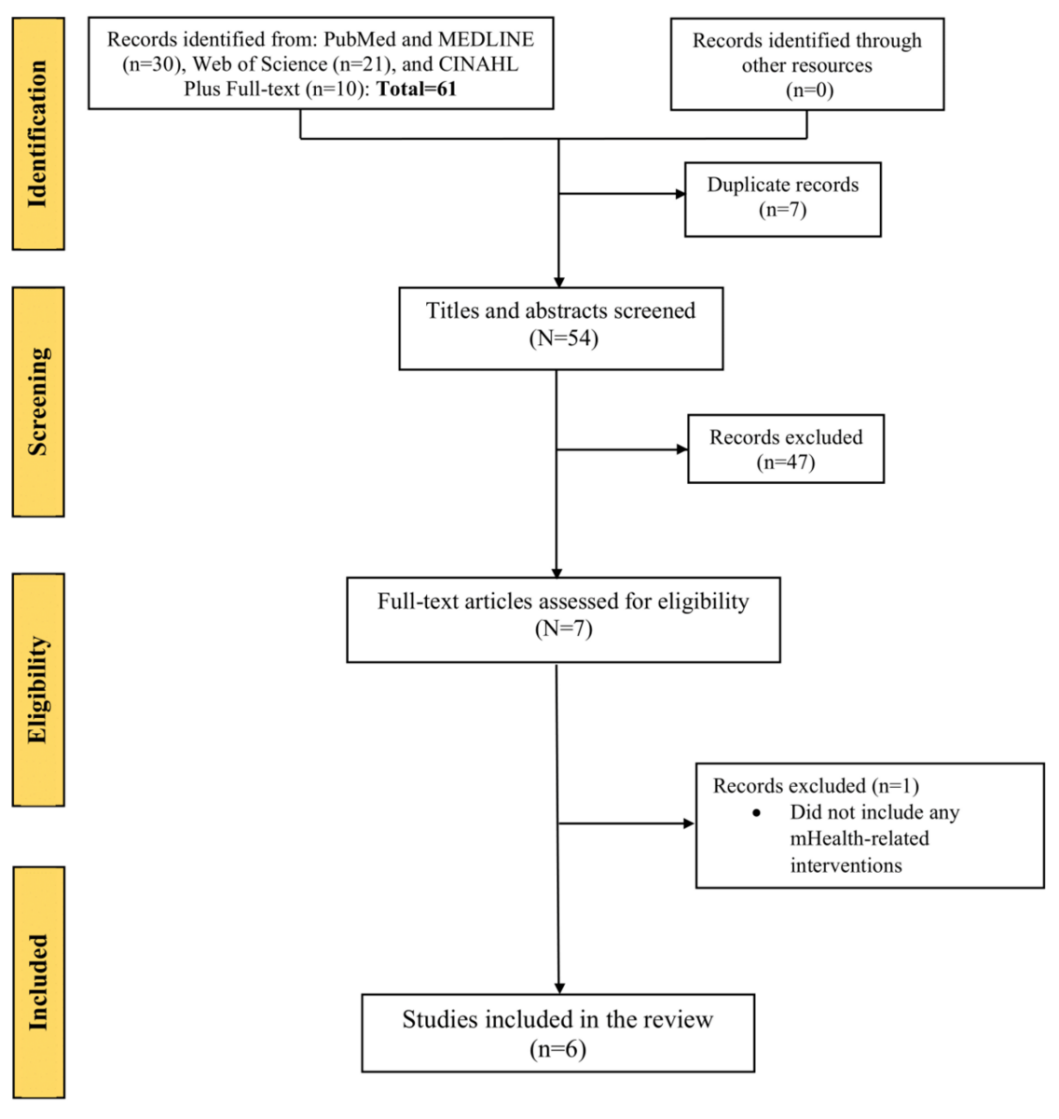
The flowchart diagram displays the selection method of qualified studies searched by a human researcher. mHealth: mobile health.

We compared the ability of humans and ChatGPT to retrieve all relevant studies. A higher recall indicates a better ability to capture all the relevant literature. Figure 1 shows the flowchart diagram of the selection of included studies based on search terms generated by human researchers. An initial literature search yielded 61 articles, including 30 from PubMed and MEDLINE, 21 from Web of Science, and 10 from CINAHL Plus Full Text. No additional records were found through other sources. After deduplication (n=7 studies), the researchers screened 54 studies, of which 47 (87%) were excluded based on the inclusion and exclusion criteria following the title and abstract screening phase. This left 7 articles for full-text screening, during which 1 article was excluded as it did not include any mHealth-related intervention. Therefore, 6 articles were included in the final analysis. It should be noted that human researchers conducted the identification, screening, eligibility, and inclusion phases.

**Figure 2:**
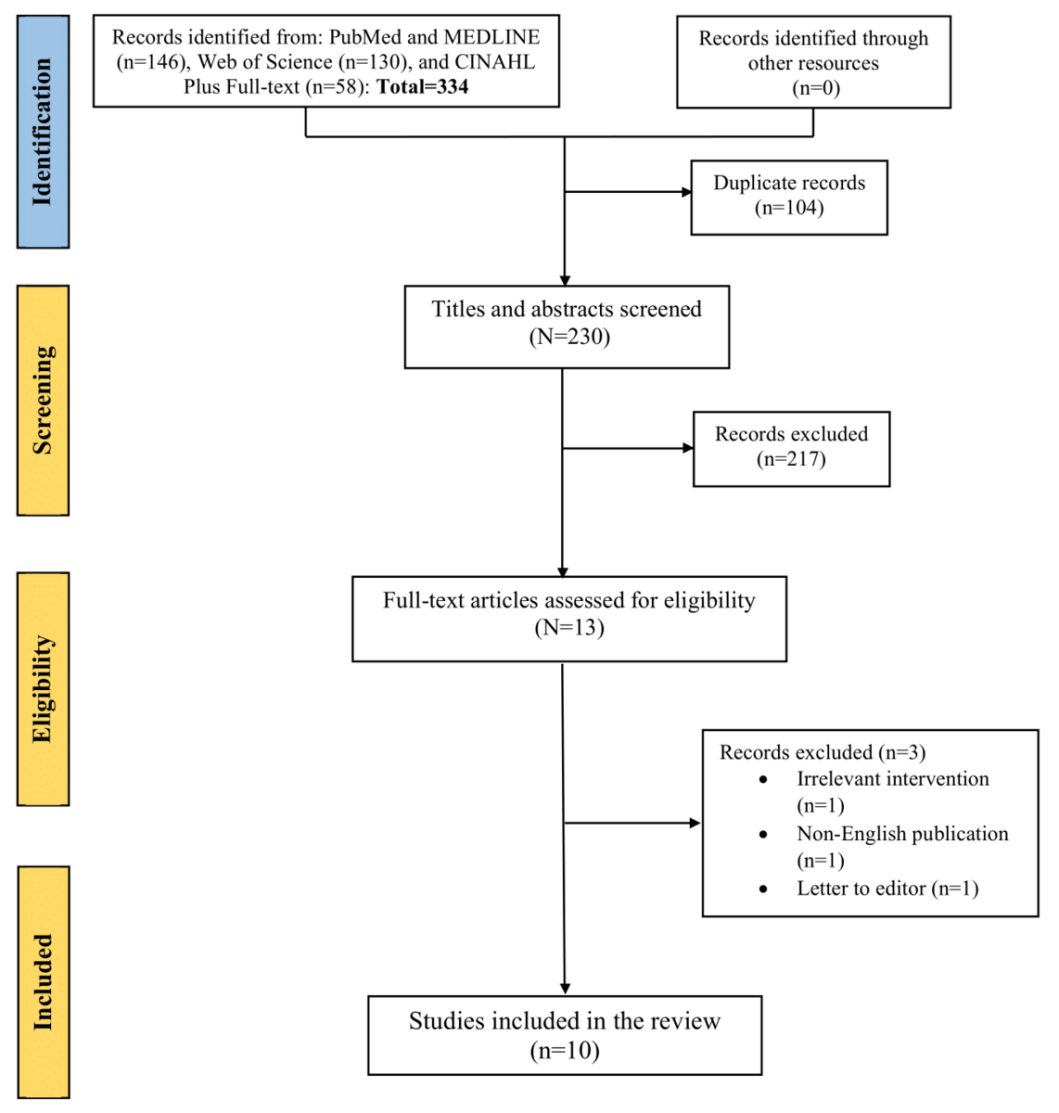
The flowchart diagram displays the selection method of qualified studies searched by ChatGPT.

Figure 2 shows the flowchart diagram of the selection of included studies based on search terms generated by ChatGPT. An initial literature search yielded 334 articles, including 146 from PubMed and MEDLINE, 130 from Web of Science, and 58 from CINAHL Plus Full Text. No additional records were found through other sources. After deduplication (n=104 studies), the researchers screened 230 studies, of which 217 (94.3%) were excluded based on the inclusion and exclusion criteria following the title and abstract screening phase. Of the 13 articles that underwent full-text screening, 3 studies were excluded because the intervention was irrelevant (n=1, 33%), the publication was not in English (n=1, 33%), and it was a letter to the editor (n=1, 33%). Finally, 10 articles were included in the final analysis. It should be noted that ChatGPT has been used only in the identification phase. The human researcher conducted the screening, eligibility, and inclusion phases.

### Description of the Included Studies

#### Studies Included From Human Searches

The analysis included 6 studies obtained from the human search (Multimedia Appendix 1). Most of these studies (3/6, 50%) were published in 2020. Among the countries where the studies were conducted, 50% (3/6) were from China, whereas 17% (1/6) of the studies each originated from Belgium, the Republic of Korea, and Sweden. In terms of study design, of the 6 studies, 3 (50%) were cohort studies, 2 (33%) were randomized controlled trials (RCTs), and 1 (17%) was a non-RCT. The sample sizes varied, with 50% (3/6) of the studies having a sample size ranging from 1 to 300 and the other 50% (3/6) of the studies having a sample size of >300. Regarding the impact of mHealth interventions on medication adherence, most included studies (4/6, 67%) reported improvements in medication adherence after the intervention [11-14]. However, in 33% (2/6) of the included studies, it was not clearly stated whether medication adherence improved after the intervention [15,16].

#### Studies Included From ChatGPT Searches

A total of 10 studies were obtained from the ChatGPT search, of which 6 (60%) studies overlapped with the human searches (Multimedia Appendix 1). Most of these studies (4/10, 40%) were published in 2020. Among the countries where the studies were conducted, 50% (5/10) of the studies were from China, whereas 10% (1/10) of the studies each originated from Belgium, the Republic of Korea, Sweden, the United States, and Pakistan. In terms of study design, most were RCTs (6/10, 60%), with 30% (3/10) of the studies being cohort studies, and 10% (1/10) of the studies being a non-RCT. The sample sizes varied, with 70% (7/10) of the studies having a sample size ranging from 1 to 300 (70%), and the other 30% (3/10) of the studies having a sample size of >300. Regarding the impact of mHealth interventions on medication adherence, most included studies (8/10, 80%) reported improvements in medication adherence after the intervention [11-14,17-20]. However, in 20% (2/10) of the included studies, it was not clearly stated whether medication adherence improved after the intervention [15,16].

### Accuracy

In our study, we used precision as a metric to assess the accuracy of both human researchers and ChatGPT in identifying relevant studies from electronic databases during the systematic review process. By comparing their precision scores, we aimed to determine which approach yielded a higher proportion of true positives (correctly identified relevant studies) and a lower rate of false positives (incorrectly identified irrelevant studies). The precision calculation formula used was as follows: precision=true positives/(true positives+false positives).

Moreover, the human researcher conducted identification, screening, eligibility, and inclusion phases, as illustrated in Figure 1. In contrast, ChatGPT was used only during the identification phase, and the human researcher conducted the screening, eligibility, and inclusion phases, as depicted in Figure 2. Therefore, we also calculated the percentage of relevance using the formula ([true positives/total studies identified from the search]×100). This approach was chosen to ensure a fair assessment, as relying solely on a formula based on true and false positives might only reflect human variability and accuracy during the screening, eligibility, and inclusion phases.

For human researchers, the precision in accurately identifying relevant studies from electronic databases was calculated as 6/(6+1)=0.86, where 6 is the number of studies included in the review (true positive) and 1 (false positive) represents the study that was incorrectly identified as relevant for inclusion in the review (did not include any mHealth-related intervention; Figure 1). This means that out of the studies deemed relevant by human researchers, 86% (6/7) were indeed appropriate for inclusion in the review, whereas 14% (1/7) were falsely identified as relevant. The percentage of relevance for the human researcher was calculated as follows: (true positives/total studies identified from the search)×100=(6/61)×100=9.8%.

Regarding ChatGPT, its precision in accurately identifying relevant studies from electronic databases was calculated as 10/(10+3)=0.77, where 10 is the number of studies included in the review (true positive) and 3 (false positive) represents the studies that were incorrectly identified as relevant for inclusion in the review (irrelevant intervention, non-English publication, and a letter to the editor; Figure 2). This indicates that out of the studies identified by ChatGPT as potentially relevant, 77% (10/13) were indeed relevant and suitable for inclusion in the review, whereas 23% (3/13) were mistakenly identified as relevant. The percentage of relevance for ChatGPT was calculated as follows: (true positives/total studies identified from the search)×100=(10/334)×100=3%.

According to our findings, the precision of human researchers was higher (precision=0.86) compared to ChatGPT (precision=0.77). This is consistent with the percentage of relevance, where human researchers (9.8%) demonstrated a higher percentage of relevance than ChatGPT (3%). These results indicate that human researchers were more effective in identifying relevant studies during the systematic review process. However, it is noteworthy that despite the lower precision and percentage of relevance, ChatGPT’s initial search yielded a significantly larger number of studies (n=334) compared to human researchers (n=61), and ultimately resulted in more studies included in the final analysis (n=10 for ChatGPT vs n=6 for human researchers). This suggests that ChatGPT’s performance was more efficient in terms of study retrieval and inclusion, although there was a 60% overlap in the studies included between both approaches.

### Efficiency

As reported in the Accuracy subsection, human researchers demonstrated higher precision in identifying relevant studies compared to ChatGPT. However, the efficiency and ability of ChatGPT to retrieve relevant studies could still hold value in the systematic review process. When considering the time required for both humans and ChatGPT to identify relevant studies, from the beginning (search term generation) to the outcome (identification of relevant studies before screening), our study found that ChatGPT substantially outperformed human researchers. ChatGPT took approximately 10 minutes, whereas human researchers spent an hour in the search term identification process using MeSH and Boolean operators before obtaining the relevant study.

In our study, we used ChatGPT to generate search terms for conducting the systematic review based on our research topic. This substantially reduced the time and effort required for initial study identification. However, it is important to note that ChatGPT’s current capabilities are limited to providing search terms, and human researchers are still required to conduct the screening of titles, abstracts, and full texts of the identified studies, using refined inclusion and exclusion criteria.

## Discussion

### Principal Findings

According to our findings, the precision of human researchers was higher compared to ChatGPT, indicating that human researchers were more accurate in identifying relevant studies during the systematic review process. Our findings are congruent with a previous study [24], which reports inaccuracies of using ChatGPT in research that requires an in-depth understanding of the literature. Likewise, Zhao et al [25] reported that the factual accuracy of ChatGPT cannot be ensured, although it has massive resources such as Microsoft and Google. In addition, a case study of using ChatGPT to conduct literature searches indicated that ChatGPT does not provide an answer to the queries that researchers ask for [26].

Despite the lower precision of ChatGPT compared to human search, a previous study reported that ChatGPT has more accurate and comprehensive relevance judgments than all other types of NLP models or techniques [27]. Moreover, our findings show that ChatGPT’s initial search yielded a significantly larger number of studies compared to human researchers and ultimately resulted in more studies being included in the final analysis despite its lower precision. This suggests that ChatGPT’s performance was more efficient in terms of study retrieval and inclusion, although there was a 60% overlap in the studies included between both approaches. Similarly, a study of ChatGPT's insights on the future of scientific publishing reports it as a valuable resource for initiating discussions [28]. However, a previous study using ChatGPT for retrieval of clinical, radiological information reported that ChatGPT provided only two-thirds of correct responses to questions [29].

Regarding the efficiency issues of using ChatGPT in identifying relevant search terms, the results of this study suggest that ChatGPT can be a useful tool for generating search terms for systematic reviews, as it can save time and effort for human researchers and potentially retrieve more relevant studies. The previous study on the use of ChatGPT Boolean query construction and refinement for systematic review showed that ChatGPT can generate queries with high precision [9]. Therefore, ChatGPT could be a valuable tool, especially for rapid reviews where time is limited and high precision is preferred over high recall [9].

Some researchers may argue that as ChatGPT has lower precision and may generate irrelevant or inaccurate terms, human researchers still need to carefully screen the studies that ChatGPT identified and verify the quality and validity of the evidence [30]. ChatGPT should be used with caution and verification and supplemented with other methods and sources to ensure the validity and rigor of the literature search [9]. Furthermore, ChatGPT’s performance may vary depending on the research topic, data availability, and input quality. Thus, future studies are needed to evaluate ChatGPT’s generalizability and reliability across different domains and contexts.

Using ChatGPT to generate search terms for systematic reviews raises some ethical questions regarding the quality and validity of the research process. Although ChatGPT may offer some advantages in terms of efficiency and comprehensiveness, it may also introduce some biases and errors that could affect the reliability and reproducibility of the systematic reviews. For example, ChatGPT may generate search terms that are irrelevant to the research topic or too broad or narrow, resulting in either missing or including studies that do not meet the inclusion criteria [31]. Moreover, ChatGPT may generate search terms that are based on its own internal knowledge and information, which may not reflect the current state of the art or the best available evidence in the field [31]. Therefore, human researchers need to carefully evaluate and validate the search terms generated by ChatGPT and document their rationale and methods for using them. In addition, human researchers need to disclose the use of ChatGPT as a tool for generating search terms and report its strengths and limitations and any potential ethical implications in their systematic review reports [31]. This would ensure that the systematic review process is transparent, accountable, and trustworthy and that the results are credible and useful for informing decision-making.

As we embark on a comparative analysis between ChatGPT and human researchers in the pursuit of identifying relevant studies within systematic reviews, particularly focused on mHealth interventions for improving medication adherence in patients with ischemic stroke, it becomes evident that several challenges and limitations underscore the intricate nature of this exploration. These challenges offer insight into the complex interplay between cutting-edge technology and the established domain expertise of human researchers, shaping the landscape in which this study unfolds.

First and foremost, the outcomes of our study are intrinsically linked to the performance of ChatGPT, an AI-driven tool that relies on its current capabilities to generate search terms. As an entity in constant evolution, ChatGPT’s performance may undergo shifts over time, potentially influencing the accuracy and efficiency with which it generates relevant search terms. Moreover, replicating the search in subsequent studies is essential due to ChatGPT’s intrinsic unpredictability. The lack of such repetition presents challenges in determining whether the observed phenomenon reflects an inherent trait of the model or is simply a random incident.

This dynamic underscores the need to interpret our findings in the context of the tool’s state during the study period. Within the realm of medical research, the intricate and evolving nature of terminology poses a formidable challenge. Although ChatGPT exhibits language generation prowess, the intricate nuances of medical terminology—constantly adapting and expanding—could potentially pose challenges to its accurate formulation of search terms. The complexity inherent to medical concepts demands a level of contextual understanding that might be challenging for an AI system.

Another pivotal consideration revolves around the potential biases embedded within ChatGPT’s training data. Drawing insights from vast data sets, ChatGPT-generated search terms might inadvertently inherit biases present in the underlying data sources. This potential bias, albeit unintentional, introduces an element of caution when relying solely on AI-generated search terms for systematic reviews. A crucial aspect of our study’s execution pertains to refining search terms. Although ChatGPT serves as a catalyst for initial search term generation, human researchers play a pivotal role in the subsequent validation and fine-tuning of these terms. This collaborative process introduces an additional layer of complexity, as human intervention becomes essential to ensure the relevance and accuracy of the generated search terms. Moreover, the resources available and the access to ChatGPT’s capabilities could introduce variability in the study’s outcomes. Depending on factors such as subscription tiers or institutional resources, the extent of ChatGPT’s contributions and, subsequently, its comparative assessment against human researchers may exhibit nuances that warrant consideration. The study’s defined scope, focused on mHealth interventions for medication adherence improvement in patients with ischemic stroke, provides a specific lens through which insights are garnered. However, this specificity inherently limits the direct transposability of findings to other medical domains or broader systematic review topics. The nuances of different research contexts might yield distinct results. Language and geographic considerations further amplify the complexity. The study predominantly engaged with studies in English, potentially omitting valuable research published in other languages or regions. This limitation underscores the need for meticulous attention to language diversity and inclusion in systematic reviews. Human researcher variability introduces a layer of subjectivity into the study. With multiple researchers contributing to search term generation, variations in expertise and individual approaches could impact the study’s outcomes. The potential for differing interpretations and formulations of search terms necessitates careful management. Publication bias, a well-known challenge in research, extends its influence into our study’s design. Both ChatGPT and human researchers might inadvertently be swayed by publication bias, where certain types of studies are more likely to be published, potentially influencing the pool of studies considered in this review.

External factors beyond the purview of our study could exert unanticipated influence. Variables such as changes in database availability, updates to search algorithms, or shifts in the research landscape might subtly shape the study’s design and outcomes, introducing an element of unpredictability. The study’s designated time frame for data collection and inclusion introduces potential time constraints and selection bias. Studies published after the search period might be inadvertently omitted, potentially impacting the completeness of the review. Although the study provides valuable insights within its specific scope, the generalizability of findings to other systematic review topics or research questions requires cautious interpretation. The intricate interplay between technology and human expertise forms the cornerstone of our study, emphasizing the necessity for a balanced and nuanced approach when leveraging ChatGPT for systematic reviews.

### The Implications of Using ChatGPT to Improve the Efficiency of Systematic Reviews

The integration of ChatGPT into the systematic review process for identifying relevant studies on mHealth interventions holds several noteworthy implications for research methodology, efficiency, and the advancement of evidence-based practices. This section explores the key implications that arise from incorporating ChatGPT as a tool to expedite and enhance the systematic review process.

One of the most immediate and impactful implications of using ChatGPT is its ability to significantly expedite the systematic review process. Traditionally, the generation of search terms for identifying relevant articles is a time-intensive task that requires meticulous crafting and refinement by human researchers. ChatGPT’s capacity to swiftly generate search terms offers an innovative solution to this bottleneck, reducing the time invested in this preliminary phase. This acceleration holds the potential to expedite the overall timeline of systematic reviews, enabling researchers to allocate more time to critical appraisal, synthesis, and analysis of selected studies.

The inherent nature of ChatGPT’s language generation capabilities allows a more diverse and expansive range of search terms. By tapping into its capacity to comprehend and generate natural language, researchers can explore a broader spectrum of keyword variations and synonyms. This expanded search scope can lead to the inclusion of studies that might have been overlooked using traditional search methods. As a result, the systematic review process becomes more comprehensive, encompassing a wider array of relevant literature.

ChatGPT’s ability to generate novel and contextually relevant search terms introduces a valuable avenue for exploratory research and hypothesis generation. Researchers can leverage ChatGPT to identify emerging trends, novel terminologies, or unconventional associations that may inform the direction of their systematic reviews. This capacity to extract insights from the vast expanse of existing literature can potentially lead to the formulation of innovative research questions and avenues for investigation.

Although ChatGPT demonstrates remarkable efficiency in generating search terms, its use necessitates a collaborative approach with human researchers. The synergy between ChatGPT’s speed and human researchers’ expertise in refining and validating search terms ensures a balanced and accurate outcome. Human researchers play a pivotal role in critically evaluating the generated search terms, refining them to align with the specific objectives of the review, and subsequently verifying the relevance of the identified articles. This collaborative interplay mitigates the risk of introducing erroneous or irrelevant studies into the review process.

In research environments with limited resources, such as time and personnel, ChatGPT offers a solution to address scalability challenges. Its ability to rapidly generate search terms can prove invaluable in scenarios where timely completion of systematic reviews is imperative. Researchers operating within resource-constrained contexts can leverage ChatGPT to conduct preliminary searches efficiently, thus optimizing the allocation of limited resources to subsequent stages of the review.

In summary, the integration of ChatGPT into the systematic review process introduces a transformative approach to enhancing efficiency and enriching the scope of literature exploration. Although its speed and breadth of search terms hold the promise of expediting the review timeline and uncovering hidden associations, the collaborative involvement of human researchers remains pivotal for ensuring accuracy, relevance, and the meticulous execution of subsequent review stages. The strategic use of ChatGPT in conjunction with traditional research practices paves the way for a new era of evidence synthesis and knowledge advancement in the field of health care interventions.

### Conclusions

Our study compares the accuracy and efficacy of human researchers and ChatGPT in providing search terms to identify articles during a systematic review on mHealth interventions for improving medication adherence in patients with ischemic stroke. Although human researchers achieved greater precision, ChatGPT’s search results exhibited lower accuracy. However, ChatGPT excelled in efficacy, taking less time to generate search terms compared to human researchers, who required more time to identify appropriate search terms. In addition, ChatGPT’s search yielded a higher number of articles compared to human researchers. Following exclusions, human researchers were left with 6 articles, and ChatGPT resulted in 10 articles after screening, 6 (60%) of which overlapped with the findings of human researchers. The use of ChatGPT in creating search terms can significantly accelerate the systematic review process, although human researchers are still essential to carry out the selection process and ensure accuracy.

## Appendix

Multimedia Appendix 1 Summary table.

## Abbreviations

AI: artificial intelligence
MeSH: Medical Subject Headings
mHealth: mobile health
NLP: natural language processing
PRISMA: Preferred Reporting Items for Systematic Reviews and Meta-Analyses
RCT: randomized controlled trial
