# Supplementary material for: Assessing the Efficacy of ChatGPT Versus Human Researchers in Identifying Relevant Studies on mHealth Interventions for Improving Medication Adherence in Patients With Ischemic Stroke When Conducting Systematic Reviews: Comparative Analysis
Source: JMIR Mhealth Uhealth. 2024 May 6;12:e51526. doi: 10.2196/51526 (PMC11106699; doi:10.2196/51526)
Supplement: Multimedia Appendix 1 [file mhealth_v12i1e51526_app1.docx]

**Multimedia Appendix 1.** Summary table.

| Included studies from human searched | | | | | Included studies from ChatGPT searched | | | | |
| --- | --- | --- | --- | --- | --- | --- | --- | --- | --- |
| Reference | Country and study design | Total sample size and target population | Intervention and objective | Main finding | Reference | Country and study design | Total sample size and target population | Intervention and objective | Main finding |
| Kamoen et al [15], 2020 | - BelgiumA pilot study of a prospective, multicenter, interventional cohort study | - 147 - IS^a^ | - Intervention: a nurse-led self-management program (educational session during hospitalization and tips and tricks concerning a healthy lifestyle through the customized platform: websites). - Objective: using a personal coach and digital platform to improve cardiovascular risk factor control in patients after IS. | - Medication adherence: Medication adherence report of 96% (NA^b^) - Other main findings: Quality of life improved (*P*<.001). Reduction in the 10-year risk of fatal cardiovascular disease (*P*<.001) | Kamoen et al [15], 2020 | Overlap with human-searched studies | Overlap with human-searched studies | Overlap with human-searched studies | Overlap with human-searched studies |
| Kim et al [11], 2020 | - Republic of Korea - Prospective, nonrandomized, interventional study | - 99 in which 61 (62%) IS and 38 (38%) hemorrhagic stroke) | - Intervention: a 12-week smartphone-based management system intervention (regular BP^c^, blood glucose, physical activity measurements, stroke education, an exercise program, a medication program, and feedback on reviewing of records by clinicians). - Objective: to develop a smartphone-based mHealth^d^ system and to evaluate its effects on health behavior management and risk factor control in patients with stroke. | - Medication adherence: Medication compliance from app record was better at visit 2 to 3 (60.9%) than at visit 1 to 2 (47.8%; *P*<.001; Y^e^) - Other main findings: Awareness of stroke, depression, and BP was enhanced when using the smartphone-based mHealth system (*P*<.001) | Kim et al [11], 2020 | Overlap with human-searched studies | Overlap with human-searched studies | Overlap with human-searched studies | Overlap with human-searched studies |
|  |  |  |  |  |  |  |  |  |  |
| Li et al [12], 2023 | - China - A secondary data analysis from a retrospective cohort study | - 188 (65 patients paired with 123 controls) - IS and TIA^f^ | - Intervention: patients using mobile apps offered adherence promotion strategies - Objective: to evaluate the effectiveness of a secondary stroke prevention mobile app among patients with stroke/TIA through medical adherence and stroke awareness | - Medication adherence: >93.8% of patients in the mobile app group were adherent to their medications compared with 82.9% in the control group (*P*=.036; Y) - Other main findings: The intervention group was more likely to be aware of stroke warning signs (*P*=.003) and when to seek medical attention compared to the control group (*P*=.016) | Li et al [12] 2023 | Overlap with human-searched studies | Overlap with human-searched studies | Overlap with human-searched studies | Overlap with human-searched studies |
| Ögren et al [16], 2018 | - Sweden - A randomized controlled trial | - Initial 871 (usual care=438 and intervention=433); final 660 (intervention=320 and control=340) for analysis - IS, hemorrhagic stroke, and TIA | - Intervention: nurse-led, telephone-based counseling and an assessment of pharmacological treatment (a physician was consulted to assess and adjust the medical treatment when the participants did not achieve the set target for LDL-C^g^ or BP). - Objective: to evaluate whether the intervention improved BP values and LDL-C levels at 36-month follow-up compared to usual care, to evaluate whether a larger proportion of the intervention group reached set treatment targets, and to investigate trends in the effects of the intervention. | - Medication adherence: A larger proportion of the intervention group reached the treatment goal for BP (N/A) - Other main findings: The mean systolic and diastolic BP values in the intervention group were 6.1 and 3.4 mm Hg (*P*<.001), respectively, lower than the values in the control group.   The mean LDL-C level was 2.2 mmol/L in the intervention group, which was 0.3 mmol/L (*P*<.001) lower than that in controls. | Ögren et al [16], 2018 | Overlap with human-searched studies | Overlap with human-searched studies | Overlap with human-searched studies | Overlap with human-searched studies |
| Yan et al [13], 2021 | - China - A community-based, 2-arm cluster-randomized controlled trial with blinded outcome assessment | - 1299 (intervention=637 and control=662) - 87.1% IS, 12.6% hemorrhagic stroke, 0.3% not specified | - Intervention: the intervention includes supported with the SINEMA app; a smartphone app for tracking patient profiles, follow-up, visits, training, and performance indicators; and a voice message system, emphasizing medication adherence and physical activity for the patients for 12 months. - Objective: to determine whether a primary care–based integrated mobile health intervention (SINEMA) could improve stroke management in rural China. | - Medication adherence: The intervention group had improved diastolic BP (*P*<.001), health-related quality of life (*P*=.008), physical activity level (*P*<.001), adherence to statin (*P*=.003), antihypertensive medication (*P*=.039), and performance in “Timed up and go” test (*P*=.022; Y) - Other main findings: The intervention group had a -2.8 mm Hg mean difference in systolic BP compared to the control group (−7.1 vs −4.3 mm Hg, *P*=.005). | Yan et al [13], 2021 | Overlap with human-searched studies | Overlap with human-searched studies | Overlap with human-searched studies | Overlap with human-searched studies |
| Zhang et al [14], 2020 | - China - A cohort study | - 468 (intervention=101 and traditional=157 for analysis) - IS and TIA | - Intervention: a physician-assisted, WeChat-based improvement service and follow-up self-monitoring platform for medication, blood glucose, and BP. - Objective: to evaluate the WeChat-based service for IS secondary prevention designed to improve treatment adherence of discharge patients. | - Medication adherence: At 1-year follow-up, the intervention group showed a tendency for better compliance (3.0%) than the traditional group (7.0%; Y) After 2 years, living in a community-based population was a positive predictor of adherence (OR^h^=2.373; *P*=.045), whereas having a prior TIA was a negative predictor of adherence (OR=0.122; *P*=.04) - Other main findings: A lower rate of recurrent events (11.9%) was observed in the intervention group after 1 year, compared to the traditional group (13.4%). | Zhang et al [14], 2020 | Overlap with human-searched studies | Overlap with human-searched studies | Overlap with human-searched studies | Overlap with human-searched studies |
| N/A^i^ | - N/A | - N/A | - N/A | - N/A | Labovitz et al [17], 2017 | - United States - A randomized, parallel-group, 12-week study | - 28 - Adults with recently diagnosed IS receiving any anticoagulation. | - Intervention: patients were randomized to daily monitoring by the artificial intelligence platform (intervention) or to no daily monitoring (control). - Objective: to evaluate the use of an artificial intelligence platform on mobile devices in measuring and increasing medication adherence in patients with stroke on anticoagulation therapy. | - Medication adherence: Real-time monitoring has the potential to increase adherence and change behavior, particularly in patients on direct oral anticoagulant therapy (Y).   Mean (SD) cumulative adherence based on pill count was 97.2% (4.4%) for the intervention group and 90.6% (5.8%) for the control group.  Plasma drug concentration levels indicated that adherence was 100% (15/ 15) and 50% (6/12) in the intervention and control groups, respectively. |
| N/A | - N/A | - N/A | - N/A | - N/A | Kamran Kamal et al [18], 2015 | - Pakistan - A parallel-group, assessor-blinded, randomized, controlled, superiority trial | - 162 - Adult participants on multiple medications with access to a cell phone and stroke at least 4 weeks from onset (onset as defined by last seen normal) | - Intervention: the intervention group in addition to usual care received reminder SMS for 2 months that contained (1) personalized, prescription-tailored daily medication reminder(s) and (2) twice weekly health information SMS. - Objective: to design a randomized controlled trial to test the effectiveness of SMS on improving medication adherence in survivors of stroke in Pakistan. | - Medication adherence: A short intervention of customized SMS can improve medication adherence and affect stroke risk factors such as diastolic BP in survivors of stroke with complex medication regimens living in resource-poor areas (Y).   2 months, the mean medication score was 7.4 (95% CI 7.2-7.6) in the intervention group and 6.7 (95% CI 6.4-7.02) in the control group. |
| N/A | - N/A | - N/A | - N/A | - N/A | Meng-Yao et al [19], 2020 | - China - Randomized assessor-blind controlled trial | - 174 (151 for analysis, of which 75 were in the control and 76 were in the intervention group) - IS | - Intervention: CRS-HBM^j^ - Objective: to determine the impact of CRS-HBM on 5 factors ((health behavior, medication adherence, BP, disability, and stroke recurrence) on patients with hypertension who were discharged after a stroke, for an interval of 6 months after discharge. | - Medication adherence: Overall, significant improvement and an upward trend were noted for the intervention group for health behaviors, medication adherence, BP, and disability (Y). - Other main findings: No significant correlation with stroke recurrence was established due to limited incidents during the trial. |
| N/A | - N/A | - N/A | - N/A | - N/A | Li-Hong et al [20], 2018 | - China - Randomized, parallel-grouped, assessor-blinded experimental design | - 174 (158 for analysis, of which 78 were in the control and 80 in the intervention group) - Hospitalized patients with hypertension and IS | - Intervention: in-person and telephone education on health beliefs and weekly automated SMS (data taken at baseline and 3 months after discharge). - Objective: to decrease systolic BP; increase BP control rate; and improve health behaviors, including physical activity, low-salt diet, nutrition, and medication adherence. | - Medication adherence: Improved systolic BP (*P*<.001) and improved health behaviors, including physical activity, nutrition, low-salt diet, and medication adherence (Y).   Medication adherence positively correlates with the implementation of the intervention.   - Other main findings: No improvement in smoking and alcohol health behaviors |

^a^IS: ischemic stroke.

^b^NA: medication adherence was not directly reported or did not clearly state whether it improved or not.

^c^BP: blood pressure.

^d^mHealth: mobile health.

^e^Y: medication adherence improved after intervention.

^f^TIA: transient ischemic attack.

^g^LDL-C: low-density lipoprotein.

^h^OR: odds ratio.

^i^N/A: not applicable.

^j^CRS-HBM: Comprehensive Reminder System Based on Health Belief Model.
